# Tissue injury characterization by pre-contrast T1 mapping post myocardial infarction

**DOI:** 10.1186/1532-429X-15-S1-E33

**Published:** 2013-01-30

**Authors:** Shah M Azarisman, Andrew Li, Dennis T Wong, James D Richardson, Seng Keong Chua, Luay Samaraie, Samuel L Sidharta, Timothy Glenie, Kerry Williams, Ben Koschade, Karen Teo, Matthew Worthley, Stephen G Worthley

**Affiliations:** 1 Cardiovascular Unit, Royal Adelaide Hospital, Adelaide, SA, Australia

## Background

Myocardial scar and edema can be assessed by late gadolinium enhancement (LGE) and T2W cardiac magnetic resonance (CMR) respectively, but each has important limitations. T1-mapping has emerged as an alternative method to characterize acute ischemic injury and contemporary mapping sequences make this clinically feasible. We assessed the T1 relaxation time in myocardial segments exhibiting varying degrees of ischemic injury in patients after acute MI.

## Methods

T2W, T1-mapping (using Shortened Modified Look-Looker Inversion recovery sequence) and LGE imaging was performed 24-72 hours after MI on a 1.5T scanner. Assessment of acute segmental damage, in a 16-segment AHA model, was performed on matched short axis slices. Mean segmental T1 values were calculated for infarcted, adjacent/edema, microvascular obstruction (MVO) or remote segments as defined by LGE.

## Results

A total of 368 segments from 23 patients were analyzed, though 33 segments (8.97%) were excluded due to artifacts on T1-mapping. Mean segmental T1 values differed significantly according to the extent of ischemic injury: Infarcted 1038±47 ms, edema 935±33 ms, MVO 685±249ms and remote segments 855±40ms (all p<0.05 compared to each other and global mean).

## Conclusions

Pre-contrast T1 mapping can assess the extent of myocardial damage early after acute MI, with qualitatively delineation of the different components of injured myocardium. T1-mapping may be an important adjunct to LGE and T2W for identification of reversible myocardial injury.

## Funding

Cardiovascular Investigation Unit,

Royal Adelaide Hospital,

North Terrace, Adelaide,

SA 5000.

